# Cross-Cultural Adaptation and Face Validity of the Functional Mobility Assessment into Brazilian Portuguese

**DOI:** 10.1155/2020/8150718

**Published:** 2020-02-07

**Authors:** Debora C. Paulisso, Daniel M. C. Cruz, Ana Luiza C. Allegretti, Richard M. Schein, Jacqueline D. Costa, Lays C. B. Campos, Mark R. Schmeler

**Affiliations:** ^1^Department of Occupational Therapy, Universidade Federal de São Carlos, São Carlos 13565905, Brazil; ^2^Department of Occupational Therapy, University of Texas Health Science Center San Antonio, San Antonio 78229, USA; ^3^Department of Rehabilitation Science & Technology, School of Health and Rehabilitation Sciences, University of Pittsburgh, Pittsburgh 15260, USA

## Abstract

*Introduction*. The Functional Mobility Assessment (FMA) measures satisfaction with mobility devices in daily life. However, in Brazil, there is a lack of instruments which measure functional mobility.

**Objective:**

We aim to report the cross-cultural adaptation process and face validity of the FMA for use in Brazil.

**Methods:**

Two international guidelines were used in the cross-cultural adaptation process. Two independent translators translated the instrument from English to Brazilian Portuguese, and the two versions were reconciled. Two different translators back translated this reconciled version, and an expert committee analysed the resulting synthesis. For face validity, the FMA was applied with 24 participants, divided into two groups, users with disabilities (*n* = 12) and occupational therapy students (*n* = 12) and occupational therapy students (

**Results:**

The cross-cultural adaptation of the FMA was concluded, and its face validity presented that both groups understood most or completely all instrument items.

**Conclusion:**

The Brazilian version of FMA is now available in Brazilian Portuguese and has face validation. Further studies should test its psychometric properties.

## 1. Introduction

In occupational therapy, the use of assessments is essential for practice and research. Most of the tools available were previously developed in countries where English is the first language [1]. However, countries in which English is not the primary language usually use standardized instruments developed internationally. Because these instruments are created originally in a different context, they need to be adapted to the culture of the country in which they are intended to be used [2]. Studies on cross-cultural adaptation of instruments are an international issue for occupational therapy because they can allow different countries to discuss about the same measurement and to compare their results, once they are using an instrument applied internationally [3]. Cross-cultural adaptation studies are also fundamental due to instruments in the native language of the target population, which present more reliable results. Moreover, only the translation, instead of a cross-cultural adaptation, may present mistakes related to the meaning of items because concepts are deeply influenced by the local culture of a country [4].

Regarding the field of assistive technology, few standardized assessments have been translated to Brazil Portuguese. Even though it is recognized that standardized assessments are essential to measure effectiveness of an intervention, today, in this country, few instruments are available for use, such as the Quebec User Evaluation of Satisfaction with Assistive Technology (QUEST 2.0), the Assistive Technology Device Predisposition Assessment (ATD PA), the Wheelchair Skills Test Questionnaire (WSTQ version 4.3), and the Wheelchair Skills Test (WST version 4.3), for both the user and caregiver version [5–9].

Standardized instruments of functional mobility are needed in Brazil Portuguese due to the lack of instruments available. In this sense, functional mobility requires a reliable measure to assess the satisfaction of users and to document functional changes on the rehabilitation process [10]. The Functional Mobility Assessment (FMA) was developed at the University of Pittsburgh in the United States and measures levels of satisfaction of mobility device users in regard to the performance of ten daily living tasks while using the device [10, 11]. The FMA fills a gap of tools which assess mobility, as it is applicable to the entire range of mobility devices, such as wheelchairs, crutches, walkers, canes, scooters, and prosthetic limbs [11]. This instrument has been used in both research and clinical practice. The FMA is easy to use; for instance, it is short enough to be applied by practitioners, does not take long to complete, and can be administered by telephone [10–13].

The FMA adopts a Likert scale in which respondents choose from “completely agree,” “mostly agree,” “slightly agree,” “slightly disagree,” “mostly disagree,” “completely disagree,” or “does not apply” to answer each of the questions. The instrument also enables respondents to rate the priority of items, so that the first point on the scale corresponds to “minimum priority” and the tenth point corresponds to “maximum priority.” The instrument leaves blank spaces for “comments,” in which the participant is supposed to specify the reasons of disagreements with a given item, whenever that is the case [10, 11].

Considering that the FMA has the potential to encourage clinical practice and research addressing functional mobility, this study's objective was to report the cross-cultural adaptation process and face validity of the FMA Beta Version 1.0., into Brazilian Portuguese.

## 2. Materials and Methods

This is a methodological and descriptive research of a cross-cultural adaptation and face validity of an instrument. The cross-cultural adaptation was performed in seven stages, where the face validity occurred on stage 6 [14, 15]. Each stage is described in Table 1.

### 2.1. Participants

According to Pasquali [17], the purpose of performing a pretest is to ensure, even if theoretically, the instrument's face validity. This theoretical analysis is performed by a group of judges, which should include the lowest stratum of the instrument's potential users and a more sophisticated stratum. To select who would respond to the FMA, we adopted the same criteria as in the study where the lowest stratum was represented by undergraduate students of an occupational therapy program and by users of mobility aids, who would possibly face difficulties understanding some items and also provide suggestions to facilitate understanding; the highest stratum was represented by the expert committee [18].

The participants of this research were divided into two groups: Group 1: twelve (*n* = 12) people with disabilities and Group 2: twelve occupational therapy students (*n* = 12) for the pretest and face validation.

In Group 1, the participants were selected from an association of people with disabilities. The inclusion criteria consisted of using a mobility aid and being 18 years of age or older. The exclusion criteria consisted of illiterate individuals and those who scored below 18 (among those with elementary or middle school) and below 26 (among those with higher education) in the cognitive screening instrument of the Mini-Mental State Examination (MMSE) [19–21]. The MMSE examines the following items: temporal orientation, spatial orientation, registration, attention and calculation, evocation memory, language, and visual constructive capacity [21]. The maximum possible score is 30, while a cut‐off point (type out greater than) > 17 was adopted in this study, as recommended [19–22]. Face validity is important because it refers to the transparency or relevance of an instrument for both respondents and examiners [23]. In this case, participants with different diagnoses using various types of mobility aids were considered for the face validity process, comprising of the assessment of not only manual and powered wheelchairs but also canes, walkers, and lower limb prostheses. In Group 2, the inclusion criteria consisted of undergraduate students from an occupational therapy program in their 4th semester or higher. Undergraduate students were included because the instrument can be used as an interview, so both professionals and users needed to understand its use and objectives [15].

### 2.2. Instruments and Procedures

For Group 1, data were gathered in the participants' homes in a single session. For Group 2, the participants were interviewed at the Department of Occupational Therapy where the research was conducted.

The instruments applied to both groups were

1. form to characterize participants addressing sociodemographic data
2. Mini-Mental State Examination (MMSE)
3. Functional Mobility Assessment (version translated into Brazilian Portuguese)
4. face validity questionnaire developed by the primary author

The participants were interviewed regarding their level of understanding of sentences and words contained in the FMA's test version.

The face validity procedure included questions for each item of the FMA for the following question:

Did you understand the item?

- I understood completely
- I understood a lot
- I understood a little
- I did not understand

### 2.3. Data Analysis and Procedures

Analysis of data obtained in the translation, reconciliation of versions, and verification of equivalence between versions was performed through simple descriptive statistics, presenting distribution of frequencies and percentages. An agreement index < 85%, obtained by the formula AI = (*n* of agreements × 100)/*n* agreements + disagreements, was the criterion adopted to reformulate the FMA's items during the cross-cultural adaptation [24].

### 2.4. Ethical Considerations

This study was in compliance with all ethical assumptions guiding research with human subjects. The respondents signed free and informed consent forms and were ensured confidentiality of their identities and information provided. The study was approved by the Institutional Review Board (Protocol No. 939.039).

## 3. Results

The results are presented by each stage of the cross-cultural adaptation as follows.

### 3.1. Translation

The versions of the two translators (T1 and T2) into Brazilian Portuguese were analysed by the two first authors of this research seeking for a consensus. We observed that both translations were very similar but differed in technical words, for instance, T2, who was a social scientist and did know about the subject “functional mobility,” translated “powered wheelchair” as “electric wheelchair” while T1 (an occupational therapist) translated correctly to “powered wheelchair.” This stage is aimed at generating a single version in Brazilian Portuguese. The other disagreements were the following: (1) translate the abbreviation of “FMA” into Brazilian Portuguese “AMF,” (2) replace the word “stage” for “step,” (3) the word “guidelines” instead of “instruction,” (4) the sentence “Answer the following ten questions” for “Answer the next ten questions,” and (5) “Fill with a “x” in the box below each answer” for “Mark with a “x” in the space below your answer.” The final decision considered the agreement of both translators who filled a form agreeing with the final decision.

### 3.2. Back translation

The Brazilian Portuguese single version of FMA was sent by e-mail to two other independent translators (B1 and B2), who at this time were native English speakers, who were also fluent in Brazilian Portuguese. They performed the back translation of the Brazilian Portuguese into English. They were not experts in the subject of the instrument. This procedure was required to avoid the bias of translators who knew the subject of FMA and to obtain possible unexpected meanings of items translated by the first translators (T12), increasing the probability to detect imperfections [14]. The first two authors of this research analysed both back translations to create a single version in English. This consensus was made together with the two translators (B12). In this stage, we identified that the title of the instrument was back translated into “Functional Mobility Evaluation (FME)” by translator B1 and “Functional Mobility Assessment” by translator B2,” where the consensus was always to be approximate of the original version; in this case, the second option was accepted.

A single version of the back translation (B12) was produced and sent to the authors of the instrument in a table where the first column had the original version of each item of FMA and the second column, the back translated version. The aim of this comparison was to check if the original and back translation version were compatible. This was confirmed by the authors who did not disagree with any items of the back translated version. These results indicate that the Brazilian Portuguese version was like the original version of FMA.

### 3.3. Expert Committee

The analysis performed by the expert committee showed that 22 sentences obtained agreement below 85% regarding semantic equivalence, 23 sentences lacked idiomatic equivalence, 25 sentences lacked conceptual equivalence, and nine lacked cultural equivalence. These, therefore, needed to be rewritten. After implementing the suggestions provided by the experts, two sentences were added, and agreement improved among the committee members. In terms of semantic equivalence, 56 of the 60 sentences were approved, as well as 57, 60, and 55 statements that referred to idiomatic, conceptual, and cultural equivalences, respectively.

Table 2 presents qualitative data from the cross-cultural adaptation where each sentence with a low level of agreement among the experts was analysed by the two first authors of this study.

### 3.4. Face Validity

An average score of 25.83 was obtained by the users of mobility aids on the MMSE applied in the pretest. This result indicates that the participants were cognitively able to assess the Brazilian version of the FMA.

Table 3 illustrates the characterization of the participants of Group 1 users of mobility devices, where it can be observed that ten were wheelchair users.

Table 4 presents data about Group 2: occupational therapy undergraduate students, where it can be identified that they knew previous knowledge about mobility.

Table 5 show that in the total of eighteen sentences of the FMA, both groups presented good levels of understanding where it can be seen that the majority of respondents of both groups understood completely or most statements of the instrument.

## 4. Discussion

Our study presented the cross-cultural adaptation process of the FMA in Brazil. Particularly, studies focusing on the cross-cultural adaptation of instruments which assess the use of assistive devices are fundamental, first, because of the worldwide interest in the development of international standards and second, due to an emerging consensus on the role of technology in promoting health and well-being [24]. Moreover, standardized instruments, sensitive to the needs of clients, should be used to ensure the process of prescribing an assistive technology product [10, 25, 26]. In Brazil, cross-cultural adaptation of instruments is growing partly related to a need for standardization and systematization of research procedures and interventions focused on assistive technology [7].

Stage 2 (translation) showed that the technical terms were important to be reviewed to address the correct meaning. For example, “electric wheelchair” was not the correct term for “powered wheelchair,” and this change was only possible when the translations were carefully compared by the two main authors of this study. We believed that these findings had influence on Stage 3 (back translation), where there was identified minimum disagreement between the two translators. This trend of having less modifications on the back translation has been observed on cross-cultural adaptation of other instruments [1, 3, 8, 9]. We also observed that the single version of back translation, when compared to the original version of FMA, was very similar, which could be a result of the work on Stages 2, 3, and 4.

However, it is important to discuss that, in our research, the cultural context was evidenced more when the expert committee suggested changes in the translation of the FMA items. These findings demonstrate the importance of the cross-cultural adaptation once the translated version from the previous stages was not enough to make the instrument precisely into Brazilian Portuguese. A similar result was found in the adaptation of the Psychosocial Impact of Assistive Device Scale (PIADS), intended for Puerto Rican assistive technology users [27]. Fifty-eight changes were suggested by five experts for the instrument adapted to Spanish, which shows the relevance of Stage 5—expert committee—in the adaptation process of an instrument to a given language and context [27].

These results were expected in the process of cross-cultural adaptation because the specific role of experts is to revise all the translated versions and synthesize them into a single version. We believe that the implementation of the suggestions provided by the experts and by the authors of the original version of FMA, as recommended, improved comprehension of the version into Brazilian Portuguese and enriched the process [14, 15].

Such a process supports the work of researchers and practitioners, who can be able to describe the same phenomenon similarly even when applying the instrument in different cultures, ensuring that the instrument works as expected [27].

Face validity showed that users and undergraduate students (who will be future therapists) understood the instrument well. This is an interesting result because users and assistive technology practitioners need to understand the construct of the instrument in order to establish a relationship of cooperation, and, in this case, the FMA promotes collaboration between users and therapists, improving therapy results [10].

Finally, the cross-cultural adaptation was satisfactorily achieved, and the various stages, from the initial translation, synthesis and back translation, and expert committee, to face validity, enabled achieving such results, considering that these are the stages recommended to ensure the quality of the process of cross-cultural adaptations [15, 28].

## 5. Conclusions

This study presented the process of cross-cultural adaptation of the FMA into Brazilian Portuguese. This tool is semantically, idiomatically, culturally, and conceptually adapted to the Brazilian context, while its format and content are in accordance with the FMA instrument in its original version. We identified that FMA has good face validation and allows therapists to gather relevant data related to satisfaction and priorities regarding mobility devices.

Our study has limitations as well, such as a small sample size (face validity step); however, the fact that the sample comprised of two different groups (students and users) was a positive aspect and presented satisfactory comprehension of the items for both respondents. Although given that the respondents were not blinded, this could be a bias that possibly influenced on their answers.

In conclusion, the use of the FMA can support therapists to identify problems related to the satisfaction of users of mobility devices and to guide interventions focusing on seating and positioning, in order to enable satisfaction and participation of these users indoors and outdoors. The FMA can also support rehabilitation practices, contributing to evidence-based practice. Further studies should address and test the psychometric properties of concurrent and convergent validity of this assessment once they have not been examined in previous research.

## Figures and Tables

**Table 1:** Cross-cultural adaptation procedures of Functional Mobility Assessment for Brazil.

Stages	Description
Preparation (stage 1)	The study was approved by the original authors of the FMA and by the Institutional Review Board at the hosting university.

Forward translation (stage 2)	The instrument was translated from its original language (English) into the target language (Brazilian Portuguese) by two independent translators (T1 and T2). T1 was an occupational therapist, and T2 was a social scientist, both fluent in English.

Reconciliation (stage 3)	The translated versions were reconciled (T12) using a table that listed the discrepancies, which was sent to each translator via e-mail.

Back translation (stage 4)	The synthesis that resulted from the reconciliation of the first two first versions was back translated (from Brazilian Portuguese into English) by two translators whose native language was English but who were proficient in Brazilian Portuguese.

Harmonization and equivalence analysis (stage 5)	An expert committee composed of eleven experts, nine occupational therapists, and two physical therapists were contacted by e-mail. Ten had a master's degree, and four had PhDs. They filled in a form addressing professional information and analysed a table where the translated version of the FMA was divided into independent sentences. Each sentence was assessed in terms of semantic equivalence (SE), that is, whether the meanings of words were equivalent; idiomatic equivalence (IE), whether idiomatic expressions and colloquialisms were equivalent; conceptual equivalence (COE), whether the concepts are maintained in the translated version; and cultural equivalence (CUE), the cultural context must be coherent with the context of the country where the translated instrument will be used [16]. Whenever they disagreed with a sentence, the experts provided a justification and presented a suggestion to change it. As part of this stage, a face-to-face meeting was also held with two authors of the instruments in Nashville, TN, United States, when all the previous stages of the cross-cultural adaptation process were presented. The process in which sentences were analysed was repeated, but at this time, the table contained only the changes suggested by the expert committee. The Brazilian versions of FMA performed by two independent translators were reconciled and then back translated into English and compared to the original version. The Brazilian version was found to be equivalent to the original instrument, indicating that the instrument's concepts were preserved. During stage 5 (expert committee), however, the instrument was further changed. All sentences for which agreement was below 85% were revised.

Face validity (stage 6)	The face validity of the prefinal version of the FMA corresponded to stage 6 of cross-cultural adaptation, where 24 participants were divided into two groups: Group 1 (12 clients with disabilities who used any device that aided mobility) and Group 2 (12 undergraduate students from the occupational therapy program at the Federal University of São Carlos, São Carlos, Brazil).

Stage 7 (final report)	Finally, the cross-cultural adaptation was concluded with the final report, sent by e-mail to the authors of the original instrument, who approved all the previous stages.

**Table 2:** Analysis of the suggestions made by the expert committee and the final decision (*n* = 11).

Original items	Expert recommendations	Final decision
Step 1. Please answer the following 10 questions by placing an “X” in the box under the response	Expert 1: I suggest “the following 10 questions below.” Expert 2: “Step 1. Please, answer the following 10 questions marking “X” in the correct answer.	Rejected. Adding more words than the original version could confuse the respondents. For example, the word “correct” answer could make the respondents think that they were being evaluated of doing something wrong or right.

(Completely agree, mostly agree, slightly agree, etc.)	Expert 3: The item “mostly agree” could be replaced for “agree most of the time” because I believe it can facilitate the comprehension of respondents in differ from the scale “completely agree.”	Accepted.

(i.e., walking, cane, crutch, walker, manual wheelchair, power wheelchair, or scooter)	Expert 1: The word “walking” can be clarified by “my mobility is performed without an assistive device, for example, a ‘crutch.'” Expert 3: To use the term “cane” and to specify “crutch” (if axillary or elbow).	Accepted. We changed for “I walk without an assistive device.” Specified the type of crutch.

If you answer, ^∗^slightly, ^∗^mostly, or ^∗^completely disagree for any question	Expert 3: My opinion is that “disagree a little, disagree most of the time, or disagree completely in any question.”	Accepted.

My current means of mobility allows me to operate it as independently, safely and efficiently as possible	Expert 1: Replace the word “operate” for “use.” Expert 5: The verb “to operate,” perhaps, will affect the real meaning into Brazilian Portuguese. I suggest “to use with independence” or “allow me to use with independence.”	Accepted. We replaced the word “to operate” for “to use it” because in Brazilian Portuguese, “to operate” is a term more used for surgeries or industries.

(e.g., dressing, bowel/bladder care, eating, hygiene)	Expert 1: I suggest only “dressing” rather than “dressing myself” Expert 3: Suggestion of translation: “e.g., getting dressed, bower/bladder care. I disagree with the translation of the words “bowel/bladder care” for “urinate and defecate.” It is not the same meaning.	Accepted the following changes: “dressing myself” for only “dressing” and “urinate and defecate” for “bowel/bladder care.”

(e.g., uneven surfaces, dirt, grass, gravel, ramps, obstacles)	Experts 3 and 6: In any part of the original FMA is written “side walk or streets.” If you want to give examples to facilitate the comprehension, it is better to put in brackets. For instance, (i.e., irregular surfaces (side walk or streets), dirt, grass, gravel, ramps, obstacles) Expert 4: Some words were not translated, but the translation has the same meaning of the original.	Rejected. The three suggestions did not affect the meaning of the translated version, but just suggested a style of how to present the question. Because a bracket already exists with examples, we just thought adding another one could interfere on the fluency of the reader.

My current means of mobility allows me to use personal or public transportation	Expert 6: The emphasis “personal” is related to private means, in the opposite of public. In Brazil, expressions which indicate this notion are “private” and “particular.”	Accepted. Added “allows me to use my own transport or public transport.”

**Table 3:** Features of participant users of mobility devices (*n* = 12).

	*N*	%
Age (years)		
20 to 29	5	41.67%
30 to 39	2	16.67%
40 to 49	2	16.67%
50 or more	3	25%
Schooling		
Incomplete primary education	3	25%
Higher school	7	58.33%
Technical education	1	8.33%
Doctorate (in progress)	1	8.33%
Current mean of mobility		
Manual wheelchair	6	37.50%
Powered wheelchair	4	25%
Axillary crutch	2	12.5%
Elbow crutch	2	12.5%
Cane	1	6.25%
Walker	1	6.25%
Length of using the device (years)		
1	5	41.67%
6 to 10	1	8.33%
11 to 20	5	41.67%
21 to 23	1	8.33%
Device prescription		
Family/relatives	3	25%
Physiotherapist	3	25%
Physician	2	16.67%
Not informed	2	16.67%
Occupational therapist	1	8.33%
Bought without prescription	1	8.33%
Training with mobility device		
None	7	58.33%
Physiotherapist	4	33.33%

**Table 4:** Features of Group 2, occupational therapy undergraduate students (*n* = 12).

	*N*	%
Year of undergraduation		
3	8	66.67%
4	4	33.33%
Self-reported knowledge in mobility		
Very good	—	—
Good	7	58.33%
Medium	5	41.67%
Bad	—	—
Sources where they learned about mobility		
Undergraduation classes	12	57.14%
Short courses, lectures, and workshops	4	19.05%
Independent search	4	19.05%
Personal experience using mobility devices	1	4.76%

**Table 5:** Results regarding the level of understanding of Groups 1 (*n* = 12) and 2 (*n* = 12) in regard to the FMA's sentences (face validity)^∗^.

	Sentences	
	Frequency	Percentage
***Understanding index—Group 1***		
Options of answers		
I understood completely	14 of 18 sentences	77.78%
I understood most statements	4 of 18 sentences	22.22%
I understood a little	—	—
I did not understand	—	—
Total	18	100%
***Understanding index—Group 2***		
Options of answers		
I understood completely	9 of 18 sentences	50%
I understood most statements	9 of 18 sentences	50%
I understood a little	—	—
I did not understand	—	—
Total	18	100%

^∗^The frequency distribution was based on the analysis of the instrument's 18 sentences by 24 participants in each group.

## Data Availability

Data used to support the findings of this study are available from the corresponding author.
